# A descriptive pharmacokinetic/pharmacodynamic analysis of ceftazidime‐avibactam in a case series of critically ill patients with augmented renal clearance

**DOI:** 10.1002/prp2.1163

**Published:** 2023-12-27

**Authors:** Ying Xu, Jian Tang, Binbin Yuan, Xuemei Luo, Pei Liang, Ning Liu, Danjiang Dong, Lu Jin, Weihong Ge, Qin Gu

**Affiliations:** ^1^ Intensive Care Unit Drum Tower Hospital Affiliated to Nanjing University School of Medicine Nanjing Jiangsu China; ^2^ Nanjing Drum Tower Hospital Clinical College of Nanjing University of Chinese Medicine Nanjing China; ^3^ Department of Pharmacy Drum Tower Hospital Affiliated to Nanjing University School of Medicine Nanjing China

**Keywords:** augmented renal clearance, ceftazidime‐avibactam, critically ill patients, drug concentration, pharmacodynamics, pharmacokinetics

## Abstract

To describe the pharmacokinetics/pharmacodynamics (PK/PD) of a 2 h infusion of ceftazidime‐avibactam (CAZ‐AVI) in critically ill patients with augmented renal clearance (ARC). A retrospective review of all critically ill patients with ARC who were treated with CAZ‐AVI between August 2020 and May 2023 was conducted. Patients whose 12‐h creatinine clearance prior to CAZ‐AVI treatment and steady‐state concentration (Css) of CAZ‐AVI were both monitored were enrolled. The free fraction (*f*Css) of CAZ‐AVI was calculated from Css. The joint PK/PD targets of CAZ‐AVI were considered optimal when a Css/minimum inhibitory concentration (MIC) ratio for CAZ ≥4 (equivalent to 100% *f*T > 4 MIC) and a Css/C_T_ ratio of AVI >1 (equivalent to 100% *f*T > C_T_ 4.0 mg/L) were reached simultaneously, quasioptimal when only one of the two targets was reached, and suboptimal when neither target was reached. The relationship between PK/PD goal achievement, microbial eradication and the clinical efficacy of CAZ‐AVI was evaluated. Four patients were included. Only one patient achieved optimal joint PK/PD targets, while the other three reached suboptimal targets. The patient with optimal PK/PD targets achieved microbiological eradication, while the other three patients did not, but all four patients achieved good clinical efficacy. Standard dosages may not enable most critically ill patients with ARC to reach the optimal joint PK/PD targets of CAZ‐AVI. Optimal drug dose adjustment of CAZ‐AVI in ARC patients requires dynamic drug concentration monitoring.

**Abbreviations**
ARC: augmented renal clearance
AVI: avibactam
BL: β‐lactam/β‐lactamase inhibitor combination
CAZ: ceftazidime
CAZ‐AVI: ceftazidime‐avibactam
CI: continuous infusion
CLCr: creatinine clearance
CRKP: carbapenem‐resistant *Klebsiella pneumoniae*
CRPA: carbapenem‐resistant *Pseudomonas aeruginosa*
Css: steady‐state concentration
CVVHDF: continuous venovenous haemodiafiltration
DALI: The Defining Antibiotic Levels in Intensive Care Unit Patients
MDR: multidrug‐resistant
MIC: minimum inhibitory concentration
PD: pharmacodynamics
PK: pharmacokinetics
TDM: therapeutic drug monitoring

## 1 INTRODUCTION

Ceftazidime‐avibactam (CAZ‐AVI), a novel β‐lactam/β‐lactamase inhibitor combination (BL), was officially introduced in China in 2019. Its mechanism is as follows: avibactam (AVI) covalently binds to and inactivates β‐lactamase in plasma, preventing it from hydrolyzing ceftazidime, which allows ceftazidime (CAZ) to enter the cytomembrane of drug‐resistant bacteria, bind to penicillin‐binding proteins (PBPs), and inhibit the synthesis of bacterial cytopeptidoglycan, leading to bacterial cell lysis and death.^1^ CAZ‐AVI exhibits activity against *Klebsiella pneumoniae* carbapenemase (KPC) and OXA‐48,^2^ so it has been widely used to treat carbapenemase‐resistant *Klebsiella pneumoniae* and *Pseudomonas aeruginosa*.^3^, ^4^, ^5^

Currently, the pharmacokinetics/pharmacodynamics (PK/PD) target ratio of BLs in critically ill patients is relatively low. The PK/PD target of BLs is generally 1 ~ 4 times the minimum inhibitory concentration (MIC) < 100% *f*T <8 MIC. For critically ill patients, the optimal drug concentration must achieve 100% *f*T > 4 MIC.^6^, ^7^ The Defining Antibiotic Levels in Intensive Care Unit Patients (DALI) study evaluated the PK/PD target achievement of BLs in critically ill patients (*n* = 361), and the results showed that the compliance rate was only 35% when 100% *f*T > 4 MIC.^8^ The EXPAT study, involving 147 critically ill patients, was also designed to investigate the PK/PD target achievement of BLs. The results showed that the PK/PD target of 100% *f*T > MIC of epidemiological cut‐off value (MIC_ECOFF_) was achieved in 63. 3% of patients, and the compliance rate of 100% *f*T > 4 × MIC_ECOFF_ was 36.7%.^9^

Augmented renal clearance (ARC), a pathological phenomenon in which the creatinine clearance (CLCr) of the kidney increases beyond normal renal function, is currently defined as greater than 130 mL/min.^10^ ARC has been reported in 20% to 65% of critically ill patients, mainly in young patients and those with sepsis, trauma, brain trauma, or burns.^10^, ^11^, ^12^ To explore the impact of ARC on the PK/PD target compliance rate in critically ill patients, a study on the treatment of patients infected with *Pseudomonas aeruginosa* (PA) by BLs (*n* = 215) was performed. Among 11 patients treated with ceftazidime, the PK/PD target value was 70% *f*T > 4 × MIC, and the compliance rate was 0%, suggesting that the compliance rate of the PK/PD target value was low in patients who suffer ARC after taking recommended dosages.^13^

Therefore, the PK/PD compliance rate of critically ill patients with BLs is low, and it is estimated that the compliance rate may be even lower in critically ill patients with ARC. Both CAZ and AVI are excreted through the kidneys, and the instructions clearly state that the dose should be adjusted according to CLCr. However, no dose recommendation for CAZ‐AVI in critically ill patients with ARC has been published.

Therefore, this study reviewed PK/PD after CAZ‐AVI treatment in a series of critically ill patients with ARC and analyzed the relationship between PK/PD, microbial eradication and clinical efficacy, with a view to optimizing the CAZ‐AVI treatment for these patients.

## 2 METHODS

This retrospective study included a case series of critically ill patients with ARC who received CAZ‐AVI in Drum Tower Hospital affiliated with the Medical School of Nanjing University (Nanjing, Jiangsu, China) between August 2020 and May 2023. Patients were included if the 12‐h CLCr was measured before CAZ‐AVI administration and the steady‐state drug concentration (Css) was monitored during CAZ‐AVI administration. This study was approved by the Ethics Committee of Drum Tower Hospital affiliated with the Medical School of Nanjing University, and the need to obtain informed consent from individual patients was waived given the retrospective nature of the study.

Clinical data were collected from the electronic medical records system of Nanjing Drum Tower Hospital, including age, sex, location of infection, pathogen, MIC values of CAZ‐AVI, doses and duration of CAZ‐AVI treatment, clinical efficacy, microbiological eradication and 28‐d mortality. The data on CAZ‐AVI treatment included initial dose, duration, and Css. Css was measured as the trough concentration of CAZ‐AVI after at least 5–6 doses. The free fraction (*f*Css) was calculated by multiplying the total CAZ and AVI Css by 0.90 and 0.93, respectively, according to the plasma protein binding reported in the literature (10% and 7% for CAZ and AVI, respectively).^14^

Our main objective was to identify the joint PK/PD targets of CAZ and AVI. The combined PK/PD target was considered optimal when a Css/MIC ratio of CAZ ≥4 (equivalent to 100% *f*T > 4 MIC) and Css/C_T_ ratio of AVI > 1 (equivalent to 100% *f*T > C_T_ 4.0 mg/L) were reached simultaneously; if only one of the two targets was achieved, the result was considered quasioptimal; and if neither of the two targets was achieved, the result was considered suboptimal.^15^ Microbiological eradication failure was defined as the presence of the same bacterial pathogen in blood or body fluid cultures ≥7 days after initiation of CAZ‐AVI treatment.^16^

Renal function was directly assessed by measuring the patient's 12‐h urine creatinine content. The methods were as follows: 12 h urine was collected in the urine catheter, total urine volume was recorded, and urine creatinine concentration and synchronous blood creatinine concentration were measured: 12 h CLCr = urine creatinine concentration × urine volume/blood creatinine (mL/min).

## 3 RESULTS

During the study period, a total of 40 critically ill patients had 12‐h CLCr measurements before CAZ‐AVI administration and Css measured during treatment, and four patients with an ARC diagnosis were included in the study, with a mean (±SD) CLCr of 138.13 ± 9.0 mL/min (Table 1). The mean (±SD) age was 39.25 ± 12.26 years, and three out of four were males (75%). Of the four patients, two had a central nervous system infection, one had a pulmonary infection, and one had a biliary tract infection. In terms of pathogenic bacteria, two patients had carbapenem‐resistant *Klebsiella pneumoniae* (CRKP), and two patients had carbapenem‐resistant *Pseudomonas aeruginosa* (CRPA). All isolates were completely sensitive to CAZ‐AVI, with an MIC range of 2 to 8 mg/L.

**TABLE 1:** Demographic and clinical features of critically ill patients with ARC treated with continuous infusion of ceftazidime‐avibactam.

No.	Age/sex	Location of infection	Pathogen	CAZ MIC (mg/L)	AVI MIC (mg/L)	CAZ‐AVI dosage	CAZ‐AVI duration (days)	Ceftazidime fCss (mg/L)	Avibatam fCss (mg/L)	Ceftazidime *f*Css/MIC ratio	Ceftazidime *f*Css/4MIC ratio	Avibactam *f*Css/C_T_ ratio	PK/PD target attainment	CLCr (ml/min)	ME	Clinical efficacy	28‐d mortality
1	54/M	Lung	CRKP	1	4	2.5 Q8h	15	45.81	4.59	45.81	1.43	1.15	Optimal	130.63	Yes	Yes	Survival
2	24/F	Biliary tract	CRKP	8	4	2.5 Q8h	17	20.63	1.62	2.58	0.64	0.40	Suboptimal	130.33	No	Yes	Survival
3	40/M	CNS	CRPA	8	4	2.5 Q8h	11	12.07	0.64	1.51	0.38	0.16	Suboptimal	143.74	No	Yes	Survival
4	39/M	CNS	CRPA	8	4	2.5 Q8h	18	18.8	0.96	2.35	0.59	0.24	Suboptimal	147.82	No	Yes	Survival

Abbreviations: ARC, augmented renal clearance; CAZ‐AVI, ceftazidime‐avibactam; CLCr: creatinine clearance; CNS, central nervous system; CRKP, carbapenem‐resistant *Klebsiella pneumoniae*; CRPA, carbapenem‐resistant *Pseudomonas aeruginosa*; C_T_ = target concentration of 4 mg/L; fCss: free fraction; ME: microbiological eradication; MIC, minimum inhibitory concentration.

All four patients started with the recommended dose of CAZ‐AVI, 2.5 g Q8h (2 h infusion), and it was given for 11–18 days. All patients received monotherapy for CRKP or CRPA. The median (IQR) Css values of CAZ and AVI were 21.91 (15.29–43.91) mg/L and 1.38 (0.76–4.14) mg/L, respectively, and the calculated *f*Css values were 19.72 (13.75–39.52) mg/L and 1.29 (0.72–3.85) mg/L, respectively. Among the patients, only one patient with CRKP pneumonia had an optimal PK/PD target (75%) and achieved microbiological eradication, while the other three patients had suboptimal results (one patient with CRKP biliary tract infection and two patients with CRPA central nervous system infection) without microbiological eradication. However, all four patients achieved good clinical efficacy, and the overall 28‐d mortality rate was 0%. There were no CAZ‐AVI‐associated adverse events during treatment.

## 4 DISCUSSION

To our knowledge, this is the first study to describe the achievement of joint PK/PD targets by a 2 h infusion of CAZ‐AVI in critically ill patients with ARC and to evaluate its relationship to microbial eradication and clinical efficacy. Our findings showed that the combined PK/PD target was achieved in only 25% of patients (one in four patients) and was associated with microbial clearance but not with clinical efficacy.

ARC is common in critically ill patients and can increase the clearance rate of drugs excreted by the kidney, making it difficult to achieve target drug concentrations. There are no guidelines or consensus recommendations on the dosages of antibiotics for patients with ARC. An observational study that included 128 critically ill patients found that up to 51.6% had ARC during the study period, and treatment failure was significantly higher in ARC patients than in non‐ARC patients (27.3% vs. 12.9%, *p* = .04).^17^ As early as 2012, a study was performed to explore the correlation between the drug trough concentration and CLCr in the treatment of critically ill patients with BLs. The results showed that 58% of patients had 100% *f*T > MIC and only 31% had 100% *f*T > 4 MIC; only 19% of ARC patients had 100% *f*T > MIC. Multivariate regression analysis showed that CLCr was an important predictor of the inability to achieve BL drug concentration.^18^ Another single‐center, prospective, observational cohort study included 100 critically ill patients with CLCr ≥60 mL/min who were treated with imipenem, meropenem, piperacillin/tazobactam, or cefepime. The aim of their study was to assess the correlation of ARC, antibiotic concentration, and clinical failure. ARC was present in 64% of those patients, and ARC strongly predicted undetectable trough concentrations of BLs (i.e., very low concentrations, OR = 3.3, 95% CI 1.11–9.94).^19^ Another study reviewed 215 patients with ARC treated with BLs from 2009 to 2014 and found that the compliance rate of all BLs reached 53%, but as CLCr increased, the compliance rate gradually decreased; the compliance rate of 11 patients with ceftazidime 100% *f*T > 4 MIC was 0%.^13^ Some studies have shown that ARC (defined as CLCr >150 mL/min) patients treated with CAZ‐AVI can achieve the PK/PD target (50% *f*T > MIC) with Css. Therefore, the standard dose of CAZ‐AVI in ARC patients suffices, and no dosage increase is necessary.^20^, ^21^ However, for critically ill patients, the optimal antimicrobial concentration should be achieved, and the PK/PD target of CAZ‐AVI is 100% *f*T > 4 MIC for CAZ and 100% *f*T > 4.0 mg/L for AVI.^16^ In this study, only one of the four patients treated with CAZ‐AVI reached the joint PK/PD targets, so it is necessary to increase the dosage or change the medication regimen of CAZ‐AVI for critically ill patients with ARC.

Increasing the load dose, extending the infusion time or providing continuous infusion (CI) of BLs can improve PK/PD parameters and the treatment success rate.^22^, ^23^, ^24^ There are few studies on the PK/PD compliance status of CAZ‐AVI in critically ill patients and the optimization of treatment plans. The largest published clinical study to date included multidrug‐resistant (MDR)‐infected patients (*n* = 31) receiving CI CAZ‐AVI to investigate the rate of joint PK/PD target achievement and the clinical efficacy.^25^ The administration regimen of CAZ‐AVI in this study was infusion of a loading dose of 2.5 g within 2 h, followed by CI for 24 h (maintenance dose adjusted according to renal function). The results showed that up to 83.9% of patients had 100% *f*T > 4 MIC of CAZ, and in 57.1% of patients the dose was adjusted according to therapeutic drug monitoring (TDM). This study suggests that the use of CI may achieve better PK/PD targets than the conventional 2 h infusion regimen. Another study included 10 patients with kidney disease receiving CI CAZ‐AVI for carbapenemase‐producing gram‐negative bacilli. CAZ‐AVI was administered as a loading dose of 2.5 g within 2 h followed by CI for 24 h. Css/MIC of CAZ ≥4 (equivalent to 100% *f*T ≥ 4 MIC) and Css/C_T_ of AVI ≥ 1 (equivalent to 100% *f*T ≥ 4.0 mg/L) were used as the optimal joint PK/PD targets of CAZ‐AVI; quasioptimal was defined as one target being met, while suboptimal was defined as neither target being met. Among the patients, seven underwent kidney replacement therapy; eight patients reached the optimal targets, and two patients were quasioptimal.^15^ Another study reviewed eight patients undergoing continuous venovenous hemodiafiltration (CVVHDF) who were treated with CAZ‐AVI for difficult‐to‐treat gram‐negative infection. All patients were given a 2.5 g 2‐hour infusion loading dose followed by a 2.5 g 8 h CI regimen, and the optimal target was a CAZ Css/MIC ratio ≥4 and AVI Css/C_T_ ≥ 1 (C_T_ = 4 mg/L). All patients reached the optimal target, and five of them achieved microbiological eradication.^26^ Therefore, the current limited clinical studies suggest that the administration of a CAZ‐AVI loading dose and CI maintenance dose can improve the PK/PD compliance rate and clinical efficacy and provide a certain theoretical basis for optimizing the administration of CAZ‐AVI in critically ill patients.

Notably, two subjects had CLCr values of 130.33 and 130.63 mL/min, respectively, just exceeding the diagnostic criteria for ARC.^10^ However, it was confirmed that the higher the CLCr was, the lower the Css of CAZ‐AVI. In this study, CLCr >130 mL/min was used as the definition of ARC,^27^ but patients with a relatively high CLCr in practice, such as 100–129 mL/min, still need to be aware of the possibility of an insufficient dosage of CAZ‐AVI. It also should be noted that the CLCr of the patient with joint PK/PD attainment before medication was 130.63 mL/min, but it dropped rapidly to 65 mL/min on the second day, resulting in a higher Css of CAZ and AVI than that of the other three patients with ARC, suggesting that the CLCr of critically ill patients may vary greatly in clinical conditions and that dynamic monitoring of CLCr and Css of CAZ‐AVI is necessary. It should also be noted that the range of CLCr in the definition of ARC is very large and only needs to be greater than 130 mL/min, with no upper limit. Therefore, patients with ARC with different CLCr require different maintenance doses, and CLCr may change relatively widely during the treatment process, requiring dynamic TDM monitoring.

Our study had several limitations. First, there were few cases because of the relatively low incidence of critically ill patients with ARC with CAZ‐AVI treatment. Second, the study was retrospective, and changes in the CSS of CAZ‐AVI were not dynamically monitored in real time to adjust the treatment regimen. Finally, due to the small sample size, we cannot yet propose an optimized treatment regimen of CAZ‐AVI for critically ill patients with ARC.

In conclusion, limited clinical data suggest that the compliance rate of CAZ‐AVI in critically ill patients with ARC is low, and the optimal administration regimen can be provided by a loading dose followed by extended infusion or continuous infusion of maintenance doses, which can improve the compliance rate of PK/PD of CAZ‐AVI. We recommend dynamically monitoring the CLCr and the trough concentration of CAZ‐AVI in ARC patients and further adjust the maintenance doses according to PK/PD parameters.

## AUTHOR CONTRIBUTIONS

All work has been approved by all coauthors. Ying Xu and Qin Gu made substantial contributions to the conception and design of the study; the data were acquired by Binbin Yuan, Pei Liang, Danjiang Dong, Ning Liu, and Qin Gu; Weihong Ge, Lu Jin, and Xuemei Luo measured the drug concentrations of all patients; the analysis and interpretation of the data were performed by Ying Xu; Jian Tang, Ying Xu, and Qin Gu wrote the draft of the article and revised it critically for intellectual content. The final version was approved by all authors.

## FUNDING INFORMATION

This work was supported by grant KY202301‐40 from the Beijing Bethune Public Welfare Foundation of China.

## CONFLICT OF INTEREST STATEMENT

The authors declare that they have no competing interests.

## ETHICS STATEMENT

This study was approved by the Ethics Committee of Drum Tower Hospital affiliated with the Medical School of Nanjing University (ethical code: 2022‐261‐01).

## CONSENT TO PARTICIPATE

The need to obtain informed consent from individual patients was waived given the retrospective nature of the study. All methods were carried out in accordance with relevant guidelines and regulations.

## Data Availability

The data sets used and/or analyzed during the current study are available from the corresponding author upon reasonable request.
